# Location Information Quality: A Review

**DOI:** 10.3390/s18113999

**Published:** 2018-11-16

**Authors:** Champika Ranasinghe, Christian Kray

**Affiliations:** Institute for Geoinformatics, University of Muenster, 48149 Muenster, Germany; c.kray@uni-muenster.de

**Keywords:** quality of location information, location-based services, positional information, adaptation strategies

## Abstract

The quality of location information is an important factor for location-based services (LBSs). In the literature, the quality of location information has been defined in different ways based on varying sets of aspects. The objectives of this paper are to review existing literature discussing location information quality and to provide a consistent framework for describing and dealing with location information quality. In particular, we review existing literature on different aspects of location information quality and on factors that affect location sensing technologies (and thus location information quality). Based on this review, we also propose a simple model for describing location information quality and a classification of the strategies for dealing with variations in the quality of location information. Designers of location sensing systems can use this model as a standard vocabulary for describing the quality of location information. The classification of strategies can be used by developers of LBSs apps to design alternative strategies for dealing with location information quality on three levels: sensor-level, algorithm-level, and application-level, which are aligned with the Location Stack model.

## 1. Introduction

Location information plays a major role in location-based services (LBSs). Location-based services usually obtain location information from location sensing systems that use various technologies such as global navigation satellite systems (GNSS) and wireless fidelity (WiFi) [1]. The quality of location information produced by these systems can vary from being very accurate information to no information being available at all. Various factors contribute to this variation and many of them are difficult to avoid [2]. They are associated, for example, with the nature of signals being used, the environment in which the location sensing system is deployed, the devices being used, or the users of a location-based service. Variations in the quality of location information can negatively impact the users of LBSs. For example, low quality may lead to inaccurate or delayed instructions in a navigation support system. Therefore, it is important to consider location information quality when selecting location sensing systems for specific LBSs. Consequently, it is desirable for location sensing systems to report the quality of location information they produce. This requires a thorough understanding of what factors cause variations and which aspects of location information quality are affected by those variations. Furthermore, it also makes sense for LBS designers to create strategies that deal with potential problems resulting from variations in the quality of location information or strategies that adapt the service to the quality of location information.

**Figure 1:**
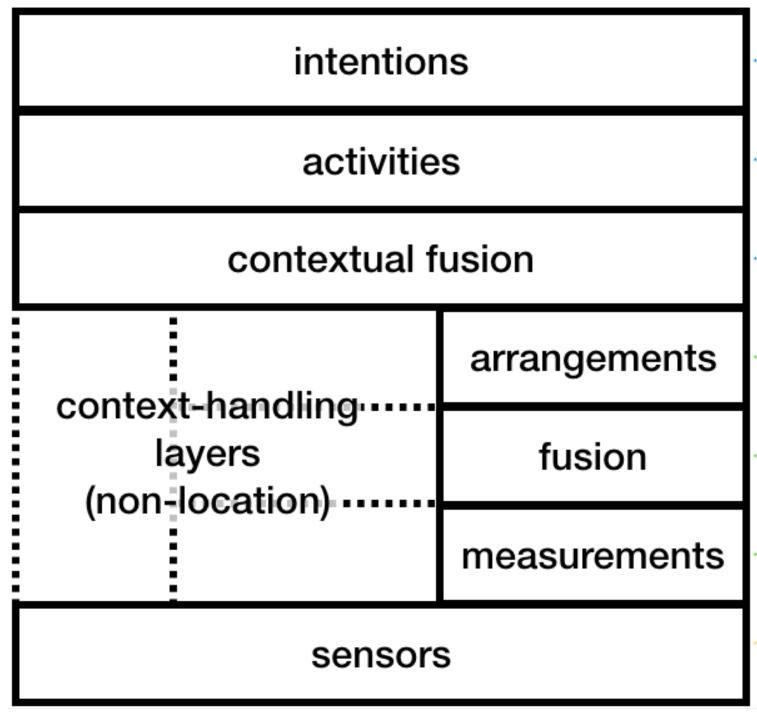
The Location Stack: A layered software engineering model for ubicomp apps developed by Hightower et al. [3].

In this article, we thus review existing literature discussing location information quality. To the best of our knowledge, this is the first systematic review of location information quality. Based on the literature, we propose a simple model for describing location information quality and a classification of existing strategies for dealing with the variations in location information quality. Designers of location sensing systems can use this model as a standard vocabulary for describing the quality of location information. The classification of strategies can be used by developers of LBSs to design alternative strategies for dealing with location information quality on three levels: sensor-level, algorithm-level, and application-level, which are aligned with the Location Stack model. Location Stack [3] is a layered standard software engineering model for LBSs. (Figure 1). It defines a seven-layered general software architecture that describes the abstractions needed by any location-aware application [3].

The remainder of the article is organized as follows: Section 2 reviews literature on different aspects of quality. Section 3 introduces a simple model for describing location information quality based on existing literature. Section 4 presents factors that cause variations in the quality of location information and how they affect the quality. Section 5 describes the classification of strategies for dealing with variations in the quality of location information. Section 7 concludes the paper.

## 2. Describing the Quality of Location Information: Related Work

In this section, we review existing literature on describing different aspects of location information quality. Previous work has identified various aspects of quality that have been connected to location information, contextual information in general or location sensing systems that could also be applied to location information.

Already during the early stages of LBSs such as GUIDE [4] and LOL@ [5] various aspects of quality of location information played a role. GUIDE used a “Bar of Connectivity” to indicate the level of reception of location information and a “Location Status Window” that displayed the last known location and the time in minutes that had elapsed since the last reading [4]. LOL@ used a tooltip text attached to the location symbol to display the accuracy of the location information [5]. Baus and Kray [6] define four different higher level qualitative categories for positional information: precise information, unprecise information, no information and false information. Aksenov et al. [7] present a middleware that encompasses different localization systems. To describe the parameters of each system, the authors use update rate and precision. In addition, each location update is characterized with an error measure and age. Dearman et al. [8] work with the accuracy of locations that they define as the “true error”, meaning the distance between the actual and predicted location. Similarly, Lemelson et al. [9] use the accuracy of a location for measuring and visualizing the expected error of an estimated position. Accuracy is also highlighted by Kjærgaard and Weckemann [10] as the estimate of positioning quality provided by GPS receivers and defined “[…] how close [a location] fix [is] to the correct, but unknown, position”.

In their work on uncertainty management, Damián-Reyes et al. [11] differentiate between uncertain, ambiguous (e.g., contradictory readings from different sensors) and wrong context. Wrong context can occur due to accuracy and precision of the used instruments, lack of information or lapsed information (time factor). Ranganathan et al. [12] use the following metrics to “[…] measure the quality of location information […]”. They use resolution as “[…] the region that the sensor says the mobile object is in” and assign a confidence value to the information that a person is within a certain area returned by a sensor. The temporal domain is covered with the concept of “freshness” meaning the time elapsed since a sensor reading (“temporal degradation”). They assign those metrics to sensors for location measurements and use those metrics to compute a probability value for each location. In the LOC8 system [13] a quality matrix for sensed location data and location sensors is applied. It contains granularity (as “[…] the smallest spatial element perceivable”), sampling frequency of a sensor, coverage (defined as “[…] the extent of space in which an entity’s position can be sensed”) and accuracy and precision pairs with accuracy as the probability that the true location is within certain distance (precision) around a sensed location. Kray and Kortuem [14] propose a quality of service model for an adaptive positioning system where requests can be made with the required quality of information. They do not only include location but also the user’s orientation and motion in the service. As parameters, their approach includes recency (time), the maximum deviation (expressed as distance in meters) as well as a required confidence regarding the location, orientation and motion. Wu et al. [15] identified five quality metrics for indoor positioning systems: accuracy and precision, coverage and its resolution, latency in making location updates, building’s infrastructure impact, effect of random errors on the system such as errors caused by signal interference and reflection [15,16].

When talking about quality of location information, it is sensible to also look at how people describe the quality of contextual information in general (as location is a key contextual parameter). Judd and Steenkiste [17] use a variety of quality metadata for contextual information queried from a database, namely accuracy, confidence, update time and sample interval. In his theoretical work on imprecision, Worboys [18] states that “[d]eficiencies in data quality, all leading to different kinds of uncertainty, may be the result of several factors”. He names inaccuracy and error (“deviation from true values”), vagueness (of describing concepts), incompleteness (missing information), inconsistency (as in conflicts) and imprecision (limited granularity). Ye et al. [19] propose a “[…] model to represent semantics of uncertainty in different levels […]”. They use accuracy and precision pairs for contextual information while “[p]recision defines the range and accuracy and the percentage of how often the accuracy is achieved”. They use the metrics incompleteness, imprecision, conflict and freshness to quantify uncertainty and use Bayes Theorem for computing a confidence value for contextual information. Others introduce similar models for sensed context information including information quality attributes: Gray et al. [20] use coverage, resolution, accuracy, repeatability (“stability of measure over time”), frequency and timeliness. Another model for handling imperfect contextual information is suggested by Henricksen and Indulska [21]: They identify “[…] four types of imperfect context information” where a property can be unknown, ambiguous, imprecise and erroneous.

When talking about the quality of *location information*, a connected and very relevant concept is the quality of *location systems*. This concept is related to the quality of the overall location sensing system (see Section 4.1) such as robustness and scalability. Therefore, we briefly review literature that talks about the quality of *location systems*. Gu et al. [22] describe eight aspects that can be used to evaluate Indoor Positioning Systems (IPSs): security and privacy, cost, performance, robustness and fault tolerance, complexity, user preference, commercial availability and, limitations. Wu et al. [15] identified five quality metrics for indoor positioning systems: accuracy and precision, coverage and its resolution, latency in making location updates, building’s infrastructure impact, the effect of random errors on the system such as errors caused by signal interference and reflection. Liu et al. [23] used accuracy, precision, complexity, scalability, robustness and cost to benchmark indoor wireless location systems. Alarifi et al. [16] defined six quality matrices to describe the performance of indoor location sensing systems. These include accuracy, availability, coverage area, scalability, cost and privacy. Mainetti et al. [24] compared location sensing systems using accuracy, coverage, cost, complexity and applicative environment. Brena et al. [25] used accuracy, coverage, and cost to compare the quality of location sensing systems. Ruiz et al. [26] identified accuracy, precision, robustness, complexity, scalability, and cost as parameters that can be used to benchmark location sensing systems. Song et al. [27] used range, accuracy, localization algorithm used and cost to compare location systems. Xiao et al. [28] defined accuracy, reliability, robustness as requirements of device-free localization. Deak et al. [29] compared location systems using accuracy, precision, complexity, scalability and cost. The above parameters were used to describe or compare the quality of *location sensing systems* rather than the quality of *location information*. These two concepts are closely connected. For example, the accuracy or precision of a location sensing system is the accuracy or the precision of location information produced by it. Another example parameter that shows the connectivity of the quality of location systems and the quality of location information is “robustness”. A robust positioning system functions well even when problematic situations such as when sensors are stolen or malfunction [22,23]. When sensors are stolen or malfunction, it has an impact on the quality of the location information produced. Therefore, these two concepts, *quality of location information* and *quality of location systems* not necessarily but can be connected. This paper talks about the *quality of location information* which is the data aspect and not about the *quality of location systems*. Table 1 summarizes the parameters used in existing literature to describe the *quality of location information* and *quality of location sensing system*.

Based on the above review, we can observe that the same term is used to describe two different aspects. For example, *precision* is used to describe accuracy, precision or granularity (resolution). Furthermore, different terms are used to refer to the same aspect. For example, *recency* is described using terms such as time elapsed since the last reading, age, lapsed information, recency, timeliness and, freshness. In the following section, we therefore propose a simple model for describing the quality of location information that consistently and clearly defines key aspects of the domain. We later use this model to analyze how different factors affect the quality of location information.

## 3. A Model for Describing Location Information Quality

**Figure 2:**
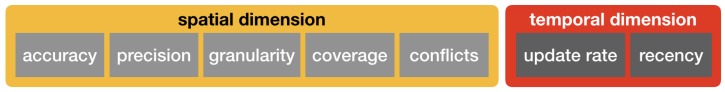
Aspects representing quality of location and orientation information along the spatial and temporal dimensions.

Taking into account existing descriptions for location information quality, we combined seven quality aspects in our model: *accuracy, precision, granularity, coverage, conflicts, update rate* and *recency* (Figure 2). From our review of related work, these seven aspects emerged as the most frequently used ones to describe quality or used to represent quality (Section 2). In addition, they represent independent dimensions of location information quality and cover practically relevant facets. We organize these aspects along two dimensions: spatial and temporal, and define them as follows.

Classically, *accuracy* describes how close a measured value is to the true value (reality), i.e., “[…] how close the fix [of a location] is to the correct, but unknown, position.” [10]. We can thus model location accuracy as a radius in meters around the measured location. We assume that somewhere inside the circle around the measured location is the true location.

*Precision* classically refers to how much repeated measurements of the same value differ under constant conditions. We can thus model precision as a radius in meters as well. When the user did not move and a new measurement is acquired, it describes how much location information deviates from the previously measured value.

Stevenson et al. [13] describe *granularity* as the smallest spatial element that is perceivable, e.g., at city block level or sub-meter level. For location, this value can be given in meters. For cell-based localization systems, cell size may define the granularity [33], whereas for continuous localization systems (e.g., GPS) it relates to the maximum achievable accuracy and/or precision.

*Coverage* can be modeled as “[…] extent of space in which an entity’s position can be sensed” [13]. For example, in the case of GPS, urban canyons may prevent localization in some areas [34]. Potentially, partial sensor failures, occlusion effects or interference could cause such behavior.

Conflicting [19] or ambiguous [11] information might lead to uncertainty about the user’s location or orientation and can result from contradictory readings from different sensors [11,35]. The mobile devices through which a location-based service is provided are usually equipped with multiple technologies for location determination. For example, the sensors in a typical smartphone allow for network-based positioning with WiFi or cellular networks and multiple GNSS. Those different sensor systems can report contradictory location readings.

While the above aspects capture spatial aspects of positional information, we also identified two main aspects of the temporal dimension: update rate and recency. The *update rate* [7], (also known as frequency [13] or sample interval [17]) describes how often a sensor measures a property. We model it as how often a new location measurement is acquired or as how many times per second the location is sampled. The term *’recency’* (also known as “freshness” [12] or “update time” [14]) can be modeled as how much time has elapsed since the last sensor reading. The less recent location information is, the more uncertainty can arise, which directly affects how well approaches such as dead reckoning work.

## 4. Factors Affecting the Quality of Location Information

This section reviews what factors cause variations in the quality of location information and how they affect it. We first present a brief overview of the technologies and methods used for localization as they have a connection to the quality variations. We then map how each factor causing variations in the quality affect different aspects of quality in the model described in Section 3. Finally, we briefly review existing strategies for mitigating the impact of these variations.

### 4.1. Location Sensing Systems

Location-based services obtain location information from various location sensing systems. These location sensing systems use some kind of technology to sense some measurements and then use localization methods to infer location from these measurements. This section briefly describes these technologies, measurements and methods used by location sensing systems.

#### 4.1.1. Localization Technologies

Location-based services rely on location sensing systems that use various technologies either individually or in combination. These technologies include, GNSS, mobile networks [36], WiFi [1,37], inertial sensors [38,39,40], infrared (IR) light [41], visible light communication (VLC) [42,43], ultrasound [44,45], audible sound [46], radio frequency identification (RFID) [47], bluetooth (BT) [48], ultra wideband (UWB) [16,49], Zigbee [50], vision (image/video) based techniques [51,52,53,54] and frequency modulation (FM) [55]. Frequently, several technologies are combined. For example, Liu et al. [56] combine WiFi and inertial sensors. Yassin et al. [57] provide a detailed review on the fusion of various localization technologies.

#### 4.1.2. Localization Measurements

Location sensing systems use various measurements to determine the location. On the one hand, these measurements largely depend on the technology used for positioning. On the other hand, a particular positioning technology can use different measurements based on various factors such as the accuracy requirements and context. The most commonly used measurements in positioning systems include: Time of Arrival (TOA also called Time of Flight (TOF), Time Difference of Arrival (TDOA), Angle of Arrival (AOA), Received signal strength (RSS), Channel state information (CSI), Id or the address of a cell/access point, acceleration, angular velocity, magnetic field and, intensity of light or sound.

***Time of Arrival*** refers to the time it takes for a signal to travel from a transmitter to a receiver. Because time can be converted to distance by multiplying it with the speed of the signal, it is a measure of the distance between the receiver and the transmitter. This distance can then be used to determine the location. Time of Arrival requires precise time synchronization between the transmitter and the receiver. ***Time Difference of Arrival*** takes the difference between the two TOA measurements between a target (the device to be located) and two (or more) synchronized destinations. This eliminates the requirement of precise time synchronization between the transmitter and the receiver. However, clock synchronization at the network level, (for example at base station level if it is a cellular network) is required. ***Angle of Arrival*** refers to the direction of incidence of a signal. This is usually measured using multi-directional antenna arrays. This technique is independent of clock synchronization. ***Received signal strength*** is a measure of the power of a received radio signal (at the receiver). ***Channel state information*** provides information about a set of channel measurements depicting the amplitudes and phases of every subcarrier of a communication link [58]. ***Cell of origin (CoO)/ID/address of a cell/access point*** refers to the cell (for example, GSM cell or an access point in a WiFi network) in closest proximity to the target. ***Acceleration*** is a measure of the acceleration forces caused either by the gravity of the Earth or by movement. Acceleration is frequently measured using accelerometers and is used to determine the speed of movement of an object or the orientation of an object in relation to the Earth’s surface. ***Angular velocity*** is the rate of change of angular displacement of a rotating body, and this can be measured using a gyroscope. This measure is useful in calculating the heading of the device. ***Magnetic field*** refers to the Earth’s magnetic field (geomagnetic field) measured by a compass. ***Ambiance or ambient measurements*** are the measurements about the surroundings such as the background sound measured by a microphone, light intensity and color measured by a camera. What measurements are selected depends mainly on the technology and computational approaches used. Generally speaking, inertial/DR systems use acceleration, angular velocity, and magnetic field. WiFi mainly uses RSS [59] but there is a trend to move towards CSI [58]. There are also WiFi-based systems that use angle measurements and time-based measurements [59,60,61]. Global navigation satellite systems (GNSS) relies on TDOA. Cellular network based approaches use a wide range of measurements depending on which localization methods they use. These could be time-based, angle based, RSS based or CoO measurements or a combination of them [62]. Visible light based positioning basically uses TOA, TDOA, AOA, CoO and RSS [42]. Radio frequency identification (RFID) usually uses RSS, TOA, and AOA (direction of arrival of a signal) [63]. Ultra wideband (UWB) is good for time-based ranging that uses TOA and TDOA [25]. However, AOA and even RSS can also be used in addition to time-based measurements [16,64]. FM usually uses RSS [65]. Bluetooth uses CoO [66] and RSS [67] measurements. Ultrasound uses TOA measurements [68] or TDOA [45]. However, use of RSS has also been proposed [69]. Infrared (IR) usually uses CoO [41].

#### 4.1.3. Localization Methods

Location sensing systems use localization methods to infer location from localization measurements. This section presents the basic localization methods in use. These include proximity sensing, lateration, angulation, fingerprinting, propagation modelling and dead reckoning.

***Proximity sensing***, also referred to as CoO or Cell-ID [23,70,71], determines the position of a target based on its presence in a given area designated by an anchor point. The location of the anchor point is taken as the location of the target. This anchor point could be, for example, a cell tower, a wireless access point, an IR reader or an RFID reader. For example, in a cellular network, the location of the base station of the cell with the highest RSSI can be taken as the location of the user.

***Lateration techniques (Distance Based Methods: Lateration/Trilateration/Multilateration)*** [70,71] use distance measurements derived from TOA, TDOA or RSS measurements to find the location of a target. In trilateration, the location of the intersection of three sphere surfaces is estimated assuming that the radius and the center of each sphere are known. The radius of the sphere is usually determined by TOA measurements. For example, in a cellular network, the location of a target mobile device can be determined by measuring TOA from three base stations assuming the locations of the base stations are known. Multilateration calculates the location of the intersection of three hyperboloids using TDOA measurements.

***Angulation/Triangulation*** [16,70] methods determine the target’s position using angular measurements (AOA) to several anchor points such as a cellular base station. Triangulation is an angulation technique that is based on the principles of plane trigonometry: if one side and two angles of a triangle are known, the other two sides and the remaining angle can be calculated. Angulation techniques usually aid the distances calculated using lateration to determine the location of a target.

***Fingerprinting*** [23,70,71] is a pattern matching technique in which some pattern of an observable feature/measurement of a target is compared with a set of pre-recorded patterns annotated with their locations to find the most probable location of the target. The observable feature that is most commonly used is the RSS. However, features such as images, projected patterns, CSI, sound or ambiance can also be used. A typical fingerprinting approach consists of two phases referred to as the offline phase and the online phase. During the offline phase, a set of measurements of the observable feature (for example RSS) are recorded at known locations and are stored in a database called the radio map. In the online phase, the same feature (for example, RSS) of the target is measured at an unknown location and is compared with the radio map to find the most similar measurement and thereby to determine the most probable location of the target.

***Propagation Modeling*** [71,72] uses signal propagation models in the location determination process. Signal propagation models model the signal strength degradation as a function of other relevant parameters such as the traveled distance and the transmission frequency [73]. For example, the well-known Okumura-Hata model models the path loss according to the distance between emitter and receiver, antenna characteristics and transmission frequency [73]. In propagation modeling based positioning, position calculations are done usually using multilateration based on distances calculated by referring the RSS to a signal propagation model [25].

In ***Dead Reckoning (DR)***, [16,39,71,74] the target’s current location is estimated relative to a known location such as the last known location or a location of an known point using the distance walked from the last known location. Measurements such as speed, acceleration and heading from sensors such as accelerometers, gyroscopes, and compasses are used to calculate the distance walked relative to a known location.

The above discussion gives an overview of the principle components of a *location sensing system*. A *localization technology* can use different *localization methods* and different *localization measurements*. Selection of measurements depends mainly on the technology and localization methods used. Generally, GNSS uses TDOA. WiFi mainly uses RSS [59] based fingerprinting but there is a trend to move towards (channel state information) CSI [58] based fingerprinting. There are also WiFi-based systems that use angle measurements and time-based measurements [59,60,61]. Cellular network based approaches use a wide range of measurements based on the localization methods they use. These could be time-based, angle based, RSS based or CoO measurements or a combination of them [62]. Visible light based positioning basically uses TOA, TDOA, AOA, CoO and RSS [42]. A description of the measurements used by visible light based positioning and example systems are available in [42]. RFID usually uses RSS, TOA, and AOA (direction of arrival of a signal) [63]. A discussion on measurements and computational approaches used in RFID is available in [63]. UWB is good for time-based ranging that uses TOA and TDOA [25]. However, AOA and even RSS can also be used in addition to time-based measurements [64]. More information on UWB positioning can be found in [16]. Bluetooth usually uses CoO [66] and RSS [67] measurements. TOA and TDOA measurements are not suitable for BT as BT lacks precise time synchronisation [67]. As BT is designed to be used in low footprint devices, directional or array antennas are rarely used making BT not a candidate for AOA based computational approaches [67]. Ultrasound uses TOA measurements [68] or TDOA [45]. However, the use of RSS has also been proposed [69]. IRusually uses CoO [41].

### 4.2. Factors Causing Quality Variations

**Figure 3:**
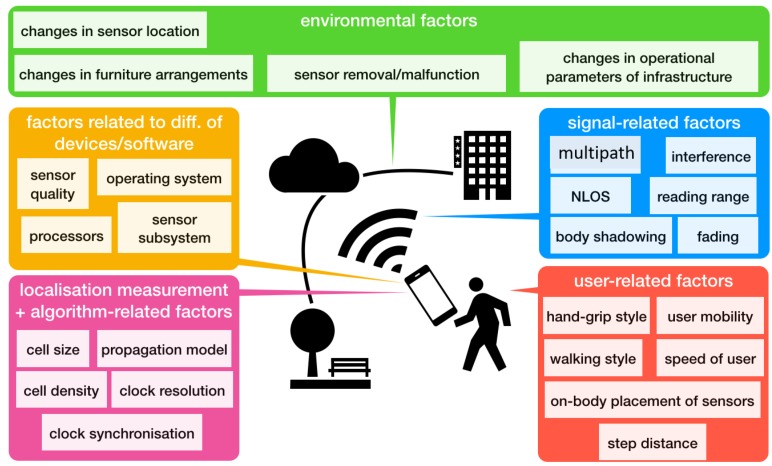
Factors that affect the quality of location information.

The quality of location information produced by location sensing systems varies from very good quality to no information at all due to various factors. These factors could be linked to the nature of the signals used by different technologies, to environmental factors, to the differences in sensors, to the localization algorithm and the nature of measurements used, or to user related differences. These categories are not necessarily independent. For example, the behavior of the signal highly depends on the environment in which the localization system is deployed. In the following, we summarize key factors along five essential dimensions that can cause variations in the quality of location information (Figure 3).

#### 4.2.1. Factors Related to the Nature of Signals

Some quality variations occur due to the inherent nature of the signals being used. These factors include multipath propagation, interferences, non-line-of-sight, body shadowing and fading. The way these factors affect the quality of location varies depending on the signals (technology) used by the location sensing system. We review these factors here and how they affect different location sensing technologies is discussed in Section 4.3.

**Figure 4:**
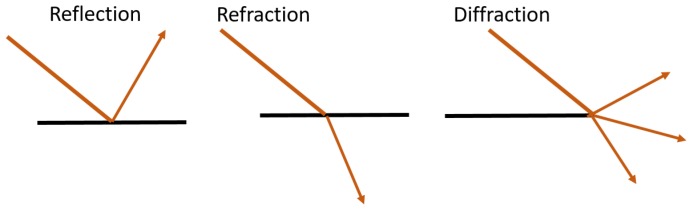
Signal propagation: reflection, refraction and diffraction.

A signal transmitted from a transmitter can arrive at the receiver as many signals in more than one path due to reflection (caused by reflective surfaces), refraction (caused by mediums with different propagation velocities) and diffraction (caused by edges) (Figure 4). These can occur at buildings, mountains, water bodies, atmospheric layers, objects, walls, ceiling, floor and metal surfaces inside buildings, etc. This is called *multipath* propagation and causes distortion, delay, loss and fading of the signal as well as loss of data [75,76]. *Interference* means that a signal transmitted by a transmitter (source) and travelling to the receiver is altered by other signals. The structure of the signal changes due to the addition of such unwanted signals. In line-of-sight (LOS) propagation, signals travel in the direct path from a source to the receiver. When this does not happen due to obstructions, refraction or reflection, we call it *Non Line of Sight (NLOS)* propagation. Significant errors in time and angle measurements occur because of NLOS propagation [77]. Some localization technologies require LOS propagation for accurate localization (e.g., IR) but in urban areas or indoor environments, the direct LOS path is often blocked due to dense buildings, bridges, concrete structures or furniture.

The human body attenuates certain types of signals [66]. This effect is called *body shadowing*. Therefore, the position of the human body when a localization measurement is taken can have an impact on the quality of location information [66]. Consequently, the density of humans [65] in a particular space can also affect the quality of location information depending on the type of technology used for localization. The reduction in the signal strength of a signal when it travels from a source to a destination is called *fading*. This happens due to various reasons such as interference from other signals, temperature, weather conditions, distance travelled, human presence, obstructions such as walls, reflection, refraction, diffraction and absorption. The quality of the location information also depends on the *reading range (communication range)* of the sensors. For example, low frequency RFID tags have a very short reading range of less than one foot whereas high frequency RFID tags have a comparatively higher reading range, often more than three feet [78].

#### 4.2.2. Factors Related to the Environment

*Nature of the environment* such as obstructions, reflective surfaces, presence of small objects and furniture arrangements affect the quality of location information. *Environmental dynamics* also have an impact on location information quality. For example, changes in the environment such as variations in the temperature, humidity, weather conditions, changes in furniture arrangements, removal or malfunctioning of the sensors (e.g., removal of a WiFi access point inside a building), or changing the location of the sensors can occur due to various reasons, e.g., to optimize aspects unrelated to location sensing. For example, WiFi access points (APs) employ optimizations such as frequency hopping to improve network throughput [65]. These optimizations cause variations in the RSSI values [65]), which in turn can have an impact on the quality of location information produced depending on the technology, measurements or the method used for localization. *Changes in operational parameters of the infrastructure* (e.g., network level changes in a base station) can also cause variations in the quality of location information [79]. Furthermore, location information quality can also vary due to *different locations having the same signal signature* [56].

#### 4.2.3. Factors Related to Differences in Devices/Software

There are differences in the quality of location produced based on device or software differences. These can include, for example, the differences in the *quality of sensors*, in the *processor* used to execute localization algorithms, and in the *sensor subsystem* that processes sensor data. On the software side, the *operating system* and its version can also make a big difference regarding location information quality. For example, GPS chips in two different phones can produce different readings at the same location. A further example relates to VLC-based positioning: image analysis can be used as a localization method and its accuracy depends, amongst other things, on the speed of the camera being used [42].

#### 4.2.4. Factors Related to Localization Measurements/Algorithm

Quality of location information also depends on the measurements used by the location sensing system. For example, time based approaches need *synchronization between clocks* and the *resolution of clocks*. Proximity based approaches depend on the *cell size* and *cell density*. Similarly, the localization method used by the location sensing system to infer the location from the measurements has an impact on the quality of location information produced. For example, systems that use *propagation models* might not accurately represent the reality and hence produce low quality location information. Fingerprinting based approaches might produce varying quality location information due to *re-calibration problems* in fingerprint databases [80,81,82].

#### 4.2.5. User Related Factors

User related factors such as the *placement of the receiver/transmitter on the body, different hand-grip styles, variance in the walking style and speed, mobility of the user* or the *orientation* cause variations in the quality of location information produced [40,83,84].

#### 4.2.6. Summary of the Factors Causing Quality Variations

The above classes represent different types of factors that affect location information quality. While we tried to make the above classes as independent as possible, there can be obvious overlaps and relationships. For example, cell density can be categorized as a algorithm related factor or an “environment” related factor; resolution of clocks can be categorized as a “device difference” or as “measurement/algorithm” related factor; Body shadowing can cause fading; Environmental dynamics can cause multipath; user walking style can affect DR based localization. Therefore, we assigned factors to those categories that we considered most fitting based on our review of related work and an analysis of how strongly each factor relates to each category.

### 4.3. Impact on Location Sensing Systems

Factors described in Section 4.2 affect different localization technologies in different ways. This section outlines these differences in more detail for the most commonly used localization technologies.

Global navigation satellite systems (GNSS) signals are often subject to fading due to reflection and refraction when they travel through the atmosphere or due to multipath reflections [83]. This factor is particularly relevant in urban canyons and indoors. Global navigation satellite systems (GNSS) signals are also affected by NLOS and by user-related issues such as different hand grip styles or body placement of the GNSS receiver [83]. Furthermore, the device type can affect accuracy: the same GNSS receiver chip installed in two different phones may produce different levels of accuracy due to the device differences [10].

Localization based on WiFi fingerprinting depends on the radio map that maps locations to WiFi signal properties. However, the RSS can vary due to many factors such as environmental changes, signal fading, orientation of the mobile device, multipath reflections, human presence (body shadowing), device used, or the presence of small objects (obstructions) [65,85]. In practice, this can result in poor or incorrect localization, e.g., when the measured RSS at a particular point differs substantially from the one recorded in the radio map due to a large number of people being present in the space. In addition, service providers can adjust the operational parameters of WiFi access points, replace WiFi routers with different models, or change the locations of access points [79]. In such situations, WiFi positioning systems based on fingerprinting will fail to provide accurate location information as the underlying radio map no longer reflects the situation in the real world. WiFi based localization is also subject to the problem of different locations having the same signal signature [56].

Cellular network based localization mainly uses propagation modelling, fingerprinting, cell ID as well as angle- and time-based methods for location calculation. These methods are also affected by various factors such as properties of the transmission medium, interference from other objects and nearby cells, as well as environmental factors [86]. It is very difficult to identify and model all the environmental factors as they are frequently site-specific and highly variable [72,87]. Approaches based on signal strength are affected by incomplete (partial) data [72,87]. Localization methods relying on cell ID are subject to variations due to cell size, cell density and environmental factors [88]. Time-based approaches (ToA, TDoA, E-OTD: enhanced observed time difference) and many angle-based approaches (AoA) require Line of Sight (LOS). Consequently, they are susceptible to NLOS. Approaches using information about a cellular network for localization (for example, fingerprinting) face problems when service providers change operational parameters at the network level.

Bluetooth is affected by multipath reflections, body shadowing, interference from WiFi and, NLOS [48,66]. Bluetooth has a low communication range (10–100 m), depending on the power class of the device. The most common one is the 10 m radius [48,89], which limits positioning granularity. Bluetooth is also subjected to fading due to its low communication range [48,66]. As Bluetooth frequently uses proximity sensing, its accuracy depends on the cell size and cell density: the smaller the cell size, the larger the number of cells, the better the accuracy [16,90]. Bluetooth signals are subject to interference as they use the same unlicensed 2.4 GHz spectrum that is widely used by other applications [16,90]. Bluetooth is also highly affected by environmental dynamics [71].

Ultra-wideband positioning is less susceptible to multipath and interference compared to other radio frequency (RF) technologies [49]. However, it is not totally immune to multipath [16,91] and is affected by interference caused by metallic materials [23]. In certain situations, such as when there is an extreme ratio of link distance to antenna height, UWB is subjected to fading and propagation delay [16]. Ultra-wideband can also interfere with nearby systems that operate in the ultra wide spectrum due to misconfiguration [16,92]. For example, in the United States, UWB operates in the frequency range of 3.1 to 10.6 GHz, which is the same used for popular communication products such as Worldwide Interoperability for Microwave Access (WiMAX) and digital TV [16].

Radio frequency identification (RFID) is affected by body shadowing, human movement, obstructions in the environment and environmental dynamics [47]. Variations in the chips and circuits could also cause quality variations [47]. In addition, RFID localization accuracy depends on the density of tags [47] and the maximal reading range of tags [24], which usually is relatively small. The number of tags that can be read simultaneously is also small [24]. Radio frequency identification (RFID) tags have different frequency ranges and behave differently depending on the frequency. The higher the frequency, the more the signal suffers from fading [47]. For example, UHF RFID technology suffers due to the absorption or reflection of RF waves in the presence of liquids [24]. High-frequency tags work reasonably well on objects made of metal and can work around goods with medium to high water content whereas low frequency tags work well in environments containing metals, liquids, dirt and mud [78,93]. Therefore, depending on the frequency used, RFID might not work at all in some environments (e.g., in environments with RF-opaque and RF-absorbent materials) [78]. In addition, the lower the frequency, the smaller the reading range of the tag [78]. Depending on the frequency used, RFID could operate without LOS [24,78]. The higher the frequency, the lower the penetrability, thus decreasing the ability to work well in NLOS environments [78].

Zigbee operates in unlicensed bands and hence is susceptible to interference from a wide range of signal types that use the same frequency [16,71]. Zigbee is also affected by reflections on metallic objects, refraction, diffraction and interference by electro-magnetic fields [50]. Vision (Image/video) based techniques require LOS [16,27]. They depend on the capabilities (processing, storage, etc.) of the underlying system and particularly the cameras being used [51].

Infrared (IR) needs LOS between the transmitter and the receiver. It is highly affected by other light sources such as sunlight and fluorescent light [22,90]. Infrared (IR) does not penetrate walls; therefore, the coverage is usually limited to smaller spaces such as a room [16,90]. Infrared (IR) communication is blocked by obstacles that block light, which includes most solid objects such as furniture, buildings or people [16,90].

Visible light communication based position is highly affected from the interference from ambient light such as sunlight [42]. Due to the high speed of light, even a small error in time measurements can cause large errors in distance estimation [42]. Therefore, the accuracy of TOA and TDOA-based VLC positioning approaches depends on the accuracy of the time measurements, which in turn depends on the resolution of clocks and the photo diode (PD) [42]. Quality of TOA-based VLC positioning approaches also depends on the response time of the receivers and rising and falling times of the light emitting diodes (LEDs) [42]. Accuracy of fingerprinting-based VLC positioning depends on how the fingerprinting database is constructed [42]. Image based VLC positioning approaches depend on the quality of the image sensors used [42]. The degree of mobility of the user also affects the quality of the location information produced [42] by VLC positioning. VLC-based positioning also suffers from multipath issues [94].

Inertial sensors frequently use Dead Reckoning (DR) for localization. In addition to variations due to the quality of the sensor hardware, DR based on inertial sensors face a number of challenges. A key issue is the accumulated drift problem, which substantially depends on the walking style of individual users [40]. The walking style is, however, highly variable between people but also for individuals [40]. In addition, the accuracy of inertial measurement units (IMUs) are highly affected by magnetic field interferences. If the inertial sensors of a phone are used, it is sensitive to the phone’s position on the body [40,95].

Ultrasound-based localization is highly affected by fading due to fluctuations in the temperature and humidity [24,68]. The speed of sound varies by approx. 0.18% per degree of Celsius. Ultrasound based positioning is also highly affected by the interference from ultrasound noise in the environment [68]. Ultrasound location sensing systems use TOA or TDOA measurements to determine location. In the case of TOA, synchronization between the emitters and receivers affect the accuracy whereas in TDOA, synchronization between receivers (or emitters depending on the architecture) affects accuracy. Furthermore, the quality of these time measurements depends on the resolution of clocks. Table 2 summarizes the above discussion.

Summarizing the discussion above, we can thus observe that the quality of positional information produced by different location sensing systems can vary due to a number of factors in real-world settings. These factors affect different aspects of quality. For example, accuracy and precision are affected by all the factors. Granularity is mainly affected by the type of technology being used. Coverage can be affected mainly by the type of technology used, multipath, interference, NLOS, fading, reading range, environmental dynamics, changes in operational parameters, device differences, quality of sensors, and placement of the transmitter/receiver in the body. Conflicts can happen due to the technology, measurements, localization methods being used and device differences or the quality of sensors. Update rate is affected by device differences and quality of sensors. Delay can occur due to multipath, NLOS, interferences and fading. Over the years, positioning technologies and localization methods have been improved to mitigate the impact of these factors to some degree. However, there still are and most likely always will be situations in which such approaches will either produce low quality location information or fail to provide location information at all. Such situations can be caused by the factors discussed in this section. The following section (Section 5) therefore discusses various strategies that have been proposed to cope with variations in the quality of location information.

## 5. Coping with Variations in the Quality of Location

Previous and current research is working on identifying ways to deal with variations in location information quality. We classify the existing strategies for dealing with the quality of location information into three classes of adaptation strategies: (a) sensor level adaptation, (b) algorithm level adaptation, (c) application level adaptation. Sensor level adaptation either improves of fuses sensors to deal with factors that affect the quality variations. Algorithm level adaptation improves or develops (computational) localization methods to improve the quality whereas application level adaptation changes the behavior of the LBS to deal with the *available* quality of location information. This section describes these three classes of strategies.

***Sensor level adaptation*****:** Sensors and other related technologies have been improved over the years, which is one way to better cope with the factors that cause variations in the quality. For example, research has resulted in improving sensor electronics and hardware, in designing better chips and processors, or in fusing several sensors. This led to sensor-level adaptation such as developing better GPS antennas, which are capable of capturing weak signals, as well as better processors, which are capable of calculating location accurately from weak signals [83]. In addition, there is research on the design and the placement of antennas to minimize the negative body-related effects [83]. The introduction of terrestrial reference stations in the context of differential GPS is another example for improving the quality of location information at the sensor level and for making the system more capable of coping with quality variations. Fusing sensors is widely used as a means for improving the quality of localization or as a means of alternative support when the main positioning technology does not work properly. For example, WiFi is combined with FM [65], sound [56], inertial sensors [85,96,97,98,99] and images [100,101] to improve the quality of location information. The review by Yassin et al. [57] provides a detailed review on the fusion of information from different technologies to improve localization accuracy.

***Algorithm level adaptation*****:** In parallel to the improvement of sensors, localization methods have also been improved or new methods have been developed to cope with variations in the quality of location information [57]. For example, approaches based on fingerprinting (e.g., using WiFi) try to cope with signal fluctuations and other issues that lead to varying quality of location by frequently re-calibrating the underlying radio map. Since manual re-calibration requires a lot of time and effort, some alternative solutions have been proposed that focus on the automatic re-calibration of the radio map using crowd-sourcing [82,96,102]. To minimize localization errors in WiFi-based localization that can occur due to multipath issues, some approaches use physical layer information as well [61,103]. In [61], the signal strength and the angle of only the direct path are extracted from multiple paths using physical layer information and human mobility data in order to minimize the multipath effect. In order to minimize the errors resulting from inaccurate and/or incomplete data in mobile network based localization, statistical modelling approaches [72] and machine learning approaches [87] have been proposed. In case fingerprinting is used in cellular networks, Otsason et al. [36] successfully used wide signal strength fingerprints that include readings from up to 29 GSM channels in addition to the six strongest cells. A good review of improvements in localization algorithms can be found in [57].

***Application level adaptation*****:** In addition to improving positional information using the approaches outlined above, it is also possible to deal with quality at the application level as a third strategy. For example, in the case of pedestrian navigation systems, it has been shown that conveying uncertainty in location information is beneficial to users [104]. Therefore, navigation systems usually use a visualization to communicate the quality of location information as a means of dealing with location uncertainty. These visualizations could be a ’bar of connectivity’ [4] to indicate the reception of location information or a ’location status window’ to show the last known location as well as the time in minutes that has elapsed since it was last measured [4]. The most common visualization method is a circular buffer around the approximate location whose size increases with decreasing quality of location information [8,104,105]. Another application level adaptation strategy used by navigation system is to start an interactive dialogue with the user to improve the quality of location information based on their answers [106].

**Figure 5:**
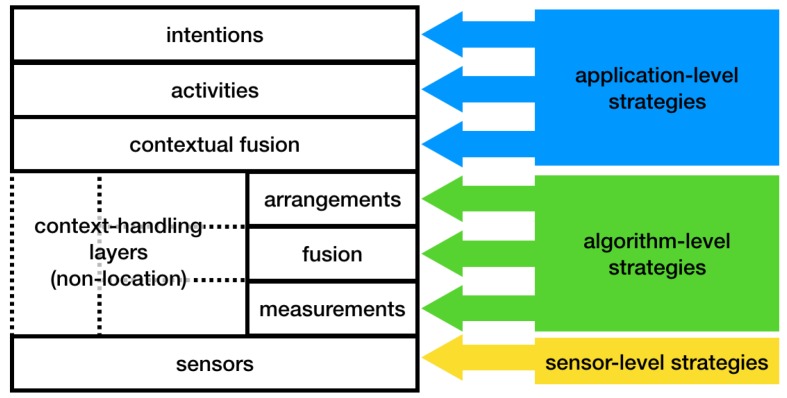
Three classes of adaptation strategies to mitigate variations in the quality of location information aligned with the layers of the Location Stack model from [3].

The three classes of adaptation strategies discussed above align well with the Location Stack [3], a standard software engineering model for LBSs (Figure 5). The Location Stack is a layered software architecture that describes the abstractions needed by any location-aware application [3]. Sensor level adaptation strategies operate on the ’sensors’ layer of the Location Stack, e.g., by improving sensors or fusing sensors. Algorithms level adaptation strategies are strategies that can be applied or implemented on the ’measurements’, ’fusion’ or the ’arrangements’ layers of the location stack. Application level adaptation strategies operate on the ’contextual fusion’, ’activities’ or the intentions’ layers of the location stack (Figure 5).

Designers of LBSs can develop adaptation strategies for the layers they consider most relevant and applicable (sensors, algorithms, application) to handle inescapable quality variations at different levels. This can include strategies at all three levels or just one or two levels based on the requirements of the application scenario. During our literature review, we found a large volume of research that pertain to sensors and algorithms layers. However, research about application level adaptation was much rarer. Location-based systems designers mainly rely on sensor and algorithm level strategies and may tend to consider variations that cannot be handled at these two levels as exceptions or bugs [107] and ignore them. Since neither type of strategy can *guarantee* the availability of accurate and timely location information, these approaches leave users vulnerable to LBS outages and failures. For example, pedestrian navigation applications use a blue circle whose size increases with decreasing location accuracy, whereas there are other quality variations such as no coverage and delay that are often ignored. This in turn can lead to confusion or wrong turns taken by the user. Further research is thus needed on how people are affected by quality variations, how they interpret them and how they cope with them. In addition, a better understanding is needed of how these questions relate to particular types of LBSs and of how to design better application level adaptation strategies.

## 6. Discussion

In this section, we first discuss the benefits of the model and the classifications we introduced in this work. We then outline the implications for future research and briefly review limitations.

### 6.1. Benefits

The model for describing the quality of location information described in Section 3 can be used as a standard vocabulary for describing various aspects of quality of location information. Current literature sometimes uses the same term for different aspects of quality as well as different terms to refer to the same aspect of quality. This can be avoided if we use a standard vocabulary for describing the quality of location information in the context of LBSs. Location sensing system designers can also use this vocabulary to report and describe the quality of location information of their systems. This will make it easier to evaluate location sensing systems for specific LBSs, to benchmark location sensing systems and to identify directions for further research. The classification of strategies used to deal with the variations in the quality of location information (Section 5) can inform the design of strategies to avoid problems due to variations of the quality of location information or to create new strategies to adapt to the quality.

### 6.2. Implications

Selecting a location sensing system for a particular application is a challenging process that can have far-reaching consequences (e.g., cost, user experience, effort). The current practice of reporting quality only in publications makes it difficult to find location sensing systems and to evaluate them based on technology, measuring, algorithms used etc. For this purpose, a central, standardized platform would be ideal to which researchers can input quality data of their location system. Other researchers can then query it and see an overview/detailed picture (e.g., What WiFi location systems have accuracy greater than X and use signal strength data from Y API’s?), or to simulate (e.g., feed RSSI data from your system and how much error produced by other systems (based on the error distribution?).

Despite improvements in sensor level or algorithm level, there will always be situations where the location sensing systems produce low quality location information. Therefore, it is essential to design application level strategies to deal with variations in the quality of location information. These strategies align with the three upper layers of the Location Stack, which can inspire their development. However, this area is still under-researched. Once location information quality is more systematically reported, the design of application-level adaptation strategies can take this data into account. For example, if factors such as body-shadowing can be more accurately modelled and more clearly linked to certain technologies, application-level strategies can be designed that motivate users to adopt poses that minimize the negative affect.

It also makes sense to conduct more research on understanding and detecting factors that affect quality and how they affect location information quality. This includes, for example, what further signal parameters represent what, how they can be mapped to quality dimensions, or whether they can be used as raw data to improve LBSs (e.g., to adapt the app interface to the available quality).

### 6.3. Limitations

In this work, we reviewed the quality of location information. A connected and very relevant concept to this is the quality of location *systems*. For example, quality aspects such as cost, scalability, or performance are quality aspects of a location system rather than of location information. This paper reviewed only the quality of location information and did not discuss quality of location systems. The former can, however, inform the latter. In other words, the *quality of location information* affects the *quality of a location system*. Therefore, aspects of location information quality can be used also as measures to describe the quality of a location system. For example, location information quality aspects such as “precision” or “update rate” are often used as measures of “performance” of a location *system*. Furthermore, quality aspects such as privacy and security could fall into either quality of location systems or quality of location information. However, our review considered these two aspects as quality of location systems rather than the quality of location information. Therefore, they were not included in our quality model for describing location information (Section 3).

The classification of strategies used to deal with the variations in the quality of location information presented in Section 5 discussed what types of strategies exist and provided some examples for each category. Our discussion of the strategies thus remained on a fairly abstract level. Each of the three classes can likely be further refined. For example, algorithm-level strategies could be subdivided into those that operate on a single type of sensor data and those that fuse multiple types. Since the focus of this article was on location information quality rather than adaptation strategies, we did not go into further detail regarding the latter. Fully analyzing and categorizing these strategies is, however, a promising endeavor for future research.

## 7. Conclusions

In this article, we made three key contributions. First, we systematically reviewed existing literature discussing location information quality. Second, we proposed a simple model for describing location information quality based on our review of existing literature on describing location information quality. Finally, we introduced a classification of strategies for dealing with the variations in location information quality.

To the best of our knowledge, this is the first systematic review of the quality of location information. It gives an overview of various aspects of location information quality, factors affecting the quality and existing approaches for dealing with the variations of the location information quality. Researchers often talk about location information quality, emphasize its importance in current and future LBSs, and develop technologies/algorithms to improve the quality of location information. However, there is still no standard way to describe and report location information quality. The model that we developed for describing location information quality consists of the seven independent, most frequently used practically relevant aspects: accuracy, precision, granularity, coverage, conflicts, update rate and recency designers of location sensing systems can use this model as a standard vocabulary for describing the quality of location information. It eliminates the problem of using the same term to refer to different aspects of quality as well as using different terms for the same aspect of quality. Having a common vocabulary to describe location information quality also makes it easier to compare location sensing systems which in turn helps selection of location systems for LBSs.

The classification of strategies for dealing with variations in the quality of location information outlines three broad classes of approaches to handle the impact of location information quality variations in LBSs (sensor level adaptation, algorithm level adaptation and application level adaptation). Designers and developers of LBSs can use this classification to design alternative strategies for dealing with location information quality on three levels. We aligned this classification with the standard software engineering model for developing LBSs, the Location Stack [3]. This makes it easier to integrate the strategies to the existing layers of the Location Stack model [3].

Overall, the review of location information quality (aspects, factors, strategies) provides the reader with an overview of concepts associated with location information quality. The model and the classification of strategies offer a consistent framework for the designers of location systems and designers of LBSs to describe and deal with the variations in the quality of location information. This helps to develop more usable and user friendly LBSs that provide better user experiences. Promising directions for future work include quantifying the impact of factors affecting location information quality, investigating their interplay and further analyzing adaptation strategies to cope with quality variations.

## Figures and Tables

**Table 1:** Parameters used in literature to describe the quality of *location information* and the quality of *location systems*.

	Parameters	Quality of Location Information	Quality of Location System
[4]	level of reception of location information, last known location, time elapsed since the last reading	✓	
[6]	precise information, unprecise information, no information, false information	✓	
[7]	error, age, update rate, precision	✓	
[11]	accuracy, precision, lack of information, lapsed information, ambiguous information (contradictory readings from different sensors)	✓	
[12]	resolution, freshness	✓	
[13]	granularity, sampling frequency of a sensor, coverage, accuracy, precision	✓	✓
[14]	recency (time), maximum deviation (expressed as distance in meters), confidence	✓	
[30]	incompleteness, accuracy, timeliness, reliability	✓	
[15]	accuracy and precision, coverage and its resolution, latency in making location updates, building’s infrastructure impact, effect of random errors on the system such as errors caused by signal interference and reflection		✓
[22]	security and privacy, cost, performance, robustness and fault tolerance, complexity, user preference, commercial availability, limitation		✓
[23]	accuracy, precision, complexity, scalability, robustness, cost		✓
[16]	accuracy, availability, coverage area, scalability, cost, privacy		✓
[24]	accuracy, coverage, cost, complexity, applicative environment		✓
[25]	accuracy, coverage, cost		✓
[26]	accuracy, precision, robustness, complexity, scalability, cost		✓
[27]	range, accuracy, localization algorithm used, cost		✓
[28]	accuracy, reliability, robustness		✓
[31]	accuracy, precision, scale, cost		✓
[32]	accuracy, complexity, cost, power consumption, usability		✓

**Table 2:** Classification of factors that affect the quality of location information.

		Localization Technology												Localization Measurement/Method							
		GNSS	WiFi	Mobile Network	BT	UWB	RFID	Zigbee	Vision/Image	IR	VLC	Inertial	Ultrasound	Cell-ID	TOA	TDOA	AOA	RSS	Fingerprinting	Prop. Modelling	DR
Signal-related	Multipath	✓[83]	✓[65,85]		✓[48,66]			✓[50]		✓[22,90]	✓[94]							✓[58]	✓[58]		
	Interference			✓[86]	✓[48,66]	✓[16,23,92]		✓[16,50,71]		✓[22,90]	✓[42]	✓[40,95]	✓[68]								✓[40,95]
	NLOS	✓[83]			✓[48,66]		✓[24,47,78,93]		✓[16,27]						✓[88]	✓[88]	✓[88]				
	Body shadowing		✓[65,85]		✓[48,66]													✓[58]			
	Fading	✓[83]	✓[65,85]		✓[48,66]		✓[24,47,78,93]											✓[58]	✓[58]		
	Reading range of sensors						✓[24,47,78,93]														
Environmental	Nature of the environment		✓[58]							✓[16,90]			✓[22]					✓[58,79]	✓[58,79]	✓[42]	
	Environmental dynamics		✓[65,85]	✓[86]	✓[71]		✓[24,47,78,93]						✓[24,68]	✓[88]				✓[58,79]	✓[58,79]		
	changes to operational parameters		✓[79]	✓[86]														✓[58,79]	✓[58,79]		
	Different locations-same signal signature		✓[56]																✓[56]		
Device/software differences	Differences in devices used	✓[10,83]	✓[65,84,85]			✓[16,23,92]			✓[42,51]		✓[42]										
	Quality of sensors	✓[83]	✓[84]			✓[16,23,92]	✓[24,47]		✓[42,51]		✓[42]										
	Quality of processors	✓[83]	✓[84]			✓[16,23,92]	✓[47]		✓[51]		✓[42]										
	Sensor-subsystem						✓[47]		✓[51]												
	OS/other software								✓[51]												
(Localization measurement/Algorithm)-related	Cell-size													✓[16,88]							
	Cell-density													✓[16,88]							
	Interference from near-by cells			✓[86]																	
	Clock synchronization																				
	Resolutions of clocks														✓[42]	✓[42]					
	Antenna resolution																✓[84]				
	Antenna array size																✓[84]				
	Outdated fingerprint databases																		✓[82]		
	Prop. model used																			✓[57]	
User-related	Hand grip styles	✓[40,83,84]																			
	Body placement of the receiver/tags	✓[83]	✓[40]	✓[40]	✓[40]		✓[40]	✓[40]				✓[40]									✓[40]
	Walking style	✓[40]	✓[40]	✓[40]	✓[40]							✓[40]									✓[40]
	Walking speed	✓[40]	✓[40]	✓[40]	✓[40]							✓[40]									✓[40]
	mobility of the user						✓[47]		✓[42]			✓[40,95]									✓[40,95]
	Orientation of the device	✓[83]	✓[65,85]																		✓[40]
